# Perturbed Brain Glucose Metabolism Caused by Absent SIRT3 Activity

**DOI:** 10.3390/cells10092348

**Published:** 2021-09-08

**Authors:** Tibor Kristian, Arman J. Karimi, Adam Fearnow, Jaylyn Waddell, Mary C. McKenna

**Affiliations:** 1 Veterans Affairs Maryland Health Center System, 10 North Greene Street, Baltimore, MD 21201, USA; afearnow@som.umaryland.edu; 2 Department of Anesthesiology and the Center for Shock, Trauma and Anesthesiology Research (S.T.A.R.), University of Maryland School of Medicine, 655 W. Baltimore St., Baltimore, MD 21201, USA; 3 Department of Pediatrics, University of Maryland School of Medicine, 655 W. Baltimore St., Baltimore, MD 21201, USA; akarimi@som.umaryland.edu (A.J.K.); jwaddell@som.umaryland.edu (J.W.); mmckenna@som.umaryland.edu (M.C.M.); 4 Program in Neuroscience, University of Maryland School of Medicine, 655 W. Baltimore St., Baltimore, MD 21201, USA

**Keywords:** SIRT3, knock out mouse, mitochondria, metabolism, [1,6-^13^C]glucose, ^13^C-NMR spectroscopy, acetylation, NAD^+^

## Abstract

Acetylation is a post-translational modification that regulates the activity of enzymes fundamentally involved in cellular and mitochondrial bioenergetic metabolism. NAD^+^ dependent deacetylase sirtuin 3 (SIRT3) is localized to mitochondria where it plays a key role in regulating acetylation of TCA cycle enzymes and the mitochondrial respiratory complexes. Although the SIRT3 target proteins in mitochondria have been identified, the effect of SIRT3 activity on mitochondrial glucose metabolism in the brain remains elusive. The impact of abolished SIRT3 activity on glucose metabolism was determined in SIRT3 knockout (KO) and wild type (WT) mice injected with [1,6-^13^C]glucose using ex vivo ^13^C-NMR spectroscopy. The ^1^H-NMR spectra and amino acid analysis showed no differences in the concentration of lactate, glutamate, alanine, succinate, or aspartate between SIRT3 KO and WT mice. However, glutamine, total creatine (Cr), and GABA were lower in SIRT3 KO brain. Incorporation of label from [1,6-^13^C]glucose metabolism into lactate or alanine was not affected in SIRT3 KO brain. However, the incorporation of the label into all isotopomers of glutamate, glutamine, GABA and aspartate was lower in SIRT3 KO brain, reflecting decreased activity of mitochondrial and TCA cycle metabolism in both neurons and astrocytes. This is most likely due to hyperacetylation of mitochondrial enzymes due to suppressed SIRT3 activity in the brain of SIRT3 KO mice. Thus, the absence of *Sirt3* results in impaired mitochondrial oxidative energy metabolism and neurotransmitter synthesis in the brain. Since the SIRT3 activity is NAD^+^ dependent, these results might parallel changes in glucose metabolism under pathologic reduction in mitochondrial NAD^+^ pools.

## 1. Introduction

Post-translational modification of protein affects activity and is one of the primary tools used by cells to adjust their response to changes in metabolic demands [1]. Regulation of protein function through acetylation is now recognized as a major factor in cellular responses initiated by pathologic situations [2]. Bioenergetic metabolism is greatly dependent on well-regulated mitochondrial function. Therefore, changes in mitochondrial protein acetylation have a significant impact on brain cells’ ability to respond to stress or pathologic conditions [3,4,5,6,7,8,9,10].

The level of protein acetylation is determined by the activity of acetyl transferases, which transfer the acetyl group from acetyl-CoA to N-ε-lysine residues of the target protein, and deacetylase activity that reverses the acetylation. Mitochondrial proteins are acetylated by mitochondrial acetyltransferase GCN5L1 [11]. The deacetylation of mitochondrial proteins is carried out by NAD^+^-dependent deacetylase sirtuin 3 (SIRT3) [12,13,14]. Although there are also other isoforms of sirtuins localized to mitochondria (SIRT4 and SIRT5), these enzymes do not contribute significantly to modulation of mitochondrial protein acetylation, but rather regulate malonylation, succinylation and ADP-ribosylation [15,16,17,18]. Therefore, mice lacking SIRT3 (SIRT3 KO) have hyperacetylated mitochondrial proteins [12,19]. Acetylation controls the activity of several mitochondrial enzymes in the TCA cycle [20,21,22], including mitochondrial complex I respiratory enzyme [23], ATP-synthase [24], the fatty acid oxidation pathway enzymes [25], and enzymes in the mitochondrial antioxidant system [26,27].

Although recent large-scale proteomics analyses revealed that every major metabolic pathway contains acetylated proteins, including glycolysis, the TCA cycle, and fatty acid metabolism, the effect of the global perturbation in mitochondrial protein acetylation on overall glucose metabolism is not known [23,25,28,29]. Here we used [1,6-^13^C]glucose administration and ex vivo ^13^C-NMR spectroscopy in SIRT3 KO mice and wild type controls to determine the role of SIRT3 on glycolytic and oxidative metabolism and neurotransmitter synthesis in the brain.

## 2. Materials and Methods

### 2.1. Animals

Adult, 3-month-old C57Bl6 wild type (WT) and SIRT3KO (B6.129S6(Cg)-Sirt^tm1.1Fwa/j^; Jackson Laboratories, Bar Harbor, ME, USA) male and female mice were used in this study. The deletion of the SIRT3 gene was confirmed by genotyping according to [12]. Only WT mice and homozygous SIRT3 KO animals showing the complete absence of SIRT3 in brain tissue [12] were used for the experiments. A total of 17 mice were used in the study: 9 WT and 8 SIRT3 KO mice. The animals were maintained in a 12-hr light/dark cycle and were housed in groups of 2 to 5 mice per cage. All mice were allowed free access to water and a maintenance diet (Teklad mouse diet, Envigo, Indianapolis, IN, USA). The animals were housed in a temperature-controlled room at 22 ± 1 °C and humidity 55 ± 15%. All cages contained bedding and nesting material (Nestlets, Ancare, Bellmore, NY, USA) for environmental enrichment. The mice were free of all viral, bacterial, and parasitic pathogens. The animal protocol was approved by the Animal Care and Used Committee of the University of Maryland, Baltimore, in accordance with the National Institutes of Health Guidelines for Care and Use of Laboratory Animals.

### 2.2. Biochemicals

The [1,6-^13^C]glucose (CLM-2717-0, 99% ^13^C enriched) and sodium 3-(trimethylsilyl)propionate-2,2,3,3-d4 (TMSP, DLM-48-1, 98%) were obtained from Cambridge Isotope Laboratories, Woburn, MA, USA. Dioxane (D111-500, 99.9%) was obtained from Thermo Fisher Scientific, Waltham, MA, USA. Perchloric acid (244252-500ML, 70%) was obtained from Sigma-Aldrich, St. Louis, MO, USA.

### 2.3. Administration of Labeled [1,6-^13^C]glucose

After the mice were weighed, they received intraperitoneal injections of [1,6-^13^C]glucose (543 mg/kg) and were euthanized by rapid decapitation 15 min post injection [30]. Anesthesia was not used, since it is well established that anesthetics decrease brain metabolism [31]. The brains were removed rapidly and snap-frozen in liquid nitrogen in under 30 s. All samples were stored at −80 °C until extraction.

### 2.4. Tissue Extraction

Frozen tissue samples were homogenized in ice-cold 7% perchloric acid (PCA) and extracted as described by Richards et al. [32]. Neutralized samples were lyophilized, and extracts were stored at −80 °C.

### 2.5. NMR Spectroscopy

Lyophilized brain samples were thawed and reconstituted in deuterium oxide (D_2_O) containing 0.02% sodium 3-(trimethylsilyl)propionate-2,2,3,3-d4 (TMSP) and 0.25% dioxane and used as internal standards for quantification of ^1^H and ^13^C spectra, respectively. The sample pH was adjusted to 6.95–7.05. ^1^H spectra and proton-decoupled ^13^C-NMR spectra were obtained on a Bruker AVANCE III 950 MHz NMR spectrometer at the NMR Center, University of Maryland, Baltimore. Fully relaxed ^1^H NMR spectra (64 scans) were acquired using a 90° pulse angle and acquisition time of 2.7 s, and a relaxation delay of 20 s. Chemical shifts for the ^1^H-spectra are reported relative to the TMSP peak at 0.0 ppm. The ^13^C-spectra were acquired at 25 °C using a 35° pulse angle, 25 KHz spectra width, and 64 K data points, using an acquisition time of 0.99 s, with a 3 s relaxation time. The average number of scans per sample was 9925. All ^13^C-spectra were corrected for nuclear Overhauser effects (nOe) and for relaxation time [30,33] by using combined nOe and a 25.7 s relaxation time as correction factors for each isotopomer [30,33]. A line broadening width of 5 Hz was used. Chemical shifts values are reported relative to the internal standard dioxane peak at 67.4 ppm. Peak assignments were made by comparison to spectra from pure standard ^13^C compounds and to literature values. All ^13^C-NMR peaks were corrected for natural abundance. Data are reported as mean ± SEM nmol ^13^C incorporated/mg protein.

### 2.6. Labeling from the Metabolism of [1,6-^13^C]glucose in the Brain

**Figure 1:**
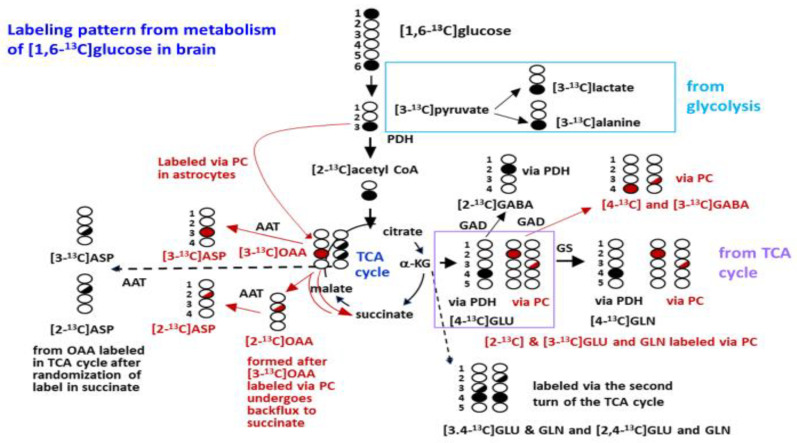
Labeling pattern from the metabolism of [1,6-^13^C]glucose in the brain. The labeling pattern from the metabolism of [1,6-^13^C]glucose via glycolysis, pyruvate dehydrogenase (PDH) and metabolism in the first turn of the TCA cycle and subsequent formation of glutamate is shown in black circles. Formation of glutamate C4 (GLU C4) from metabolism in the first turn of the TCA cycle, which is stronger in neurons but also occurs in astrocytes, and subsequent conversion to glutamine C4 (GLN C4) in astrocytes is indicated by black circles. Further metabolism of α-ketoglutarate C4 (α-KG) in the TCA cycle results in randomization of the label in the symmetrical molecule succinate and leads to formation of malate and oxaloacetate (OAA) equally labeled in the C2 and C3 positions (half-filled black circles). OAA can remain in the TCA cycle or be transaminated to form aspartate (ASP C2 and C3). Labeling of OAA from metabolism via the pyruvate carboxylase (PC) pathway in astrocytes leading is shown in red circles. Backflux of OAA C3 labeled via PC leads to partial labeling of OAA C2 (partially filled red circle). Metabolism of OAA labeled via PC leads to labeling of GLU and GLN in the C2 indicated by red circles. Some labeling in the C3 positions of GLU and GLN occurs from the backflux labeling in OAA. GABA C2 is formed from glutamate labeled via PDH in the first turn of the TCA cycle (black circles) and GABA C4 and some GABA C3 is labeled from precursors formed via PC (red circles). For simplicity, labeling from the first turn of the TCA cycle is shown and labeling of GLU and GLN from the second turn is shown. It should be noted that GLU C3 and C2 are also labeled in the second turn of the TCA cycle and GABA C3 can be formed from GLU C3 formed in neurons. Additional details of the labeling and pathways are given in the Section 2. Abbreviations: AAT, aspartate aminotransferase; GABA, Υ-aminobutyric acid; GAD, glutamic acid decarboxylase; GLU, glutamate; GLN, glutamine; GS, glutamine synthetase; OAA, oxaloacetate; PDH, pyruvate dehydrogenase complex; PC, pyruvate carboxylase. (Figure reproduced with permission from Ferreira et al. [30]).

Figure 1 represents a schematic of brain glucose metabolism, which includes the metabolic pathways participating in the ^13^C label incorporation into downstream metabolic products. Brain metabolites are rapidly labeled after intraperitoneal injection of [1,6-^13^C]glucose via activity of glycolytic enzymes and by the enzymes of the TCA cycle (Figure 1). [1,6-^13^C]Glucose is metabolized via the glycolytic pathway to form [3-^13^C]pyruvate, which can give rise to [3-^13^C]lactate and [3-^13^C]alanine. Pyruvate is primarily metabolized via pyruvate dehydrogenase (PDH) to form [2-^13^C]acetyl CoA, which enters the TCA cycle and condenses with oxaloacetate to form citrate. Metabolism in the TCA cycle leads to labeling of [4-^13^C]α-ketoglutarate in the first turn, which can be converted to [4-^13^C]glutamate (GLU C4) in neurons; this labeling is typically stronger in neurons but also occurs in astrocytes [34]. After its release by neurons, uptake of GLU C4 into astrocytes followed by metabolism via glutamine synthetase leads to the formation of the corresponding [4-^13^C]glutamine (GLN C4) in astrocytes. GLN C3 is labeled from metabolism in the second turn of the TCA cycle and can be converted to GLN C3 in astrocytes. Due to randomization of the C3 label in the symmetrical molecule succinate equal labeling in GLU C2 is formed in the second turn and subsequently GLN C2 also occurs. The GLU C4 formed in neurons is also converted directly to [2-^13^C]GABA (GABA C2). Later in the first turn of the TCA cycle [3-^13^C]oxaloacetate is formed, which can give rise to [3-^13^C]aspartate (ASP C3). In astrocytes, the metabolism of glucose occurs via the pyruvate carboxylase (PC) pathway and also via PDH. Metabolism via PC leads to the formation of [2-^13^C]glutamate and incorporation of unlabeled carbon into GLU and GLN C3. This differential labeling in GLU/GLN C2 and C3 allows for estimation of the metabolism via PC. However, due to back-flux of the TCA cycle some [3-^13^C]GLU is also formed from metabolism via PC, thus calculation of PC from the differential labeling in GLN C2/GLU C2 and GLN C3/GLU C3 provides a minimal value for labeling from metabolism via PC. Labeling in GLU C3 that occurs via PC cannot be distinguished from the GLU C3 formed in the second turn of the neuronal TCA cycle. Glutamine C2 and C3 formed via PC is released by astrocytes and taken up by neurons where it can be converted to GLU C2 and GLU C3 by phosphate-activated glutaminase. The GLU C2 formed from the precursors synthesized in astrocytes, or from labeling in the second turn of the neuronal TCA cycle, can subsequently be converted to GABA C4 in neurons. Labeling in GABA C3 is from the metabolism of the GLU C3 formed in neurons, and from the metabolism of the GLU C3 formed in astrocytes.

The anaplerotic ratio, which is a measure of labeling from metabolism via the PC pathway in astrocytes was calculated for aspartate (ASP C3-ASP C2)/ASP C3 [30,35].

### 2.7. Determination of Metabolite and Protein Concentration

The protein concentration of pellets from PCA extracts of brain was determined by the Pierce BCA micro-reagent assay. Concentrations of metabolites and amino acids were obtained from the fully relaxed ^1^H NMR spectra [36] and calculated relative to the internal standard TMSP. Correction factors determined for each of the amino acid peaks in the ^1^H spectra were applied to correct for sideband contamination at high magnetic field strength as described by Ferreira et al. [30].

### 2.8. Determination of Percent Enrichment of Labeled Metabolites

The percent enrichment of each isotopomer (isotope isomer) labeled with ^13^C from glucose metabolism was determined using the formula below. Glutamate C4 is used as an example in the formula below.

% enrichment of GLU C4 = ((GLU C4 − (0.011 × GLU C4))/concentration of Glutamate) × 100

### 2.9. Statistical Analysis

The normality of variable distribution was confirmed using the Shapiro–Wilk (IBM^®^ SPSS^®^ Statistics Version 24.0, 2016). Student’s *t*-tests were used to determine differences in the metabolite concentration in SIRT3 KO and WT brain. Student’s *t*-tests were also used to determine differences in the amount of compounds labeled from the metabolism of [1,6-^13^C]glucose, and the percent enrichment in isotopomers in SIRT3 KO and WT brain. Values are presented as means ± SEM. All values for nmol ^13^C incorporated/mg protein were corrected for natural abundance.

## 3. Results

### 3.1. Concentration of Metabolites in the Brain

**Figure 2:**
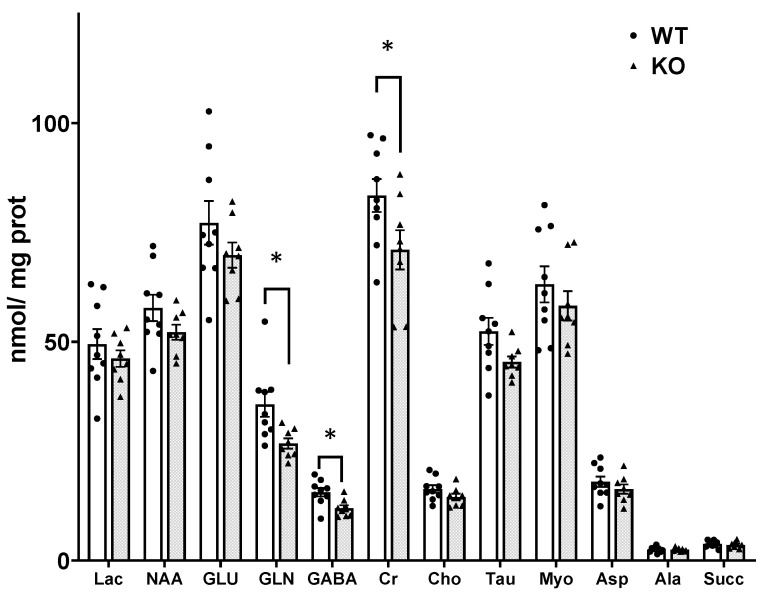
Concentration of metabolites in male and female brain from adult WT (white bars) and SIRT3 KO mice (gray bars). Individual data points are indicated by circles and triangles. Values for metabolites were obtained from ^1^H spectra of PCA extracts; concentrations were calculated in reference to the internal standard TMSP. Values for amino acids were corrected for sideband contamination as described in methods. Data are expressed as mean ± SEM. The concentrations of glutamine and GABA were lower in brains from SIRT3 KO mice compared to WT. Data from 9 SIRT3 KO and 8 WT mice were analyzed by Student’s *t*-test, * *p* < 0.05. Abbreviations: Lac, lactate; NAA, N-acetylaspartate; GLU, glutamate; GLN, glutamine; GABA, ɣ-aminobutyric acid, Cr, total creatine; Cho, choline; Tau, taurine; Myo, myo-inositol; Asp, aspartate; Ala, alanine; Succ, succinate.

High resolution ^1^H-NMR spectra of perchloric acid extracts from WT and SIRT3 KO mice brain were obtained for the determination of metabolite concentration. Male and female mice were used (3 WT females and 3 KO females). There were no differences in the concentration of N-acetylaspartate (NAA), lactate, glutamate, alanine, succinate, aspartate, choline and myo-inositol between SIRT3 KO and WT mice brain samples (Figure 2).

In contrast, the concentrations of glutamine and GABA were lower in SIRT3 KO brain compared to WT brain. Glutamine was 35.69 ± 2.83 and 26.75 ± 1.24 nmol/mg brain t(15) = 2.79, *p* = 0.013 in WT and SIRT3 KO brain, respectively. The concentration of GABA was 15.62 ± 0.98 and 11.94 ± 0.70 nmol/mg, t(15) = 2.97, *p* = 0.009, in WT and SIRT3 KO brain, respectively. Creatine (Cr) was lower in SIRT3 KO mice compared to WT mice, t(15) = 2.13, *p* < 0.049. The glial osmolyte taurine (Tau) tended to be lower in SIRT3 KO brain compared to WT mice; t(15) = 1.99, *p* = 0.064.

### 3.2. Metabolism Changes in SIRT3 KO Brain

Brain tissue was obtained from mice 15 min after intraperitoneal injection of [1,6-^13^C]glucose. The peaks for α-glucose C6, β-glucose C6 and α-glucose C1, β-glucose C1 were acquired from the ^13^C-NMR spectra and quantified relative to the internal standard dioxane as described in the methods. The concentration of unmetabolized [1,6-^13^C]glucose was similar in brains from SIRT3 KO and WT mice. The α-glucose C1 was 0.26 ± 0.05 nmol/mg protein in Sirt3 KO and 0.26 ± 0.06 nmol/mg protein in WT animals, demonstrating that the glucose uptake into the brain was not affected by the absence of Sirt3 deacetylase.

### 3.3. Glycolysis Is Not Impaired in SIRT3 KO Brain

**Figure 3:**
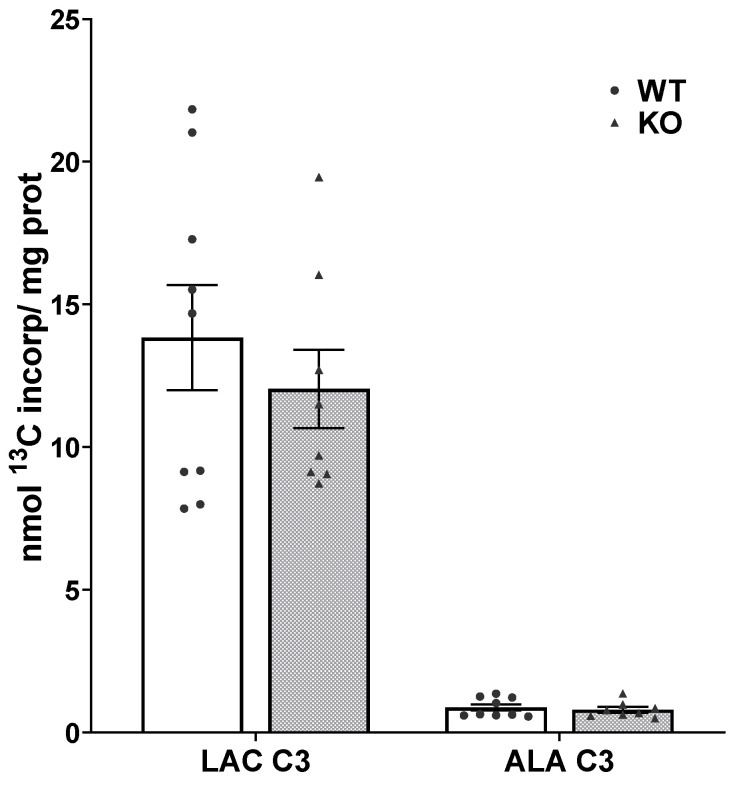
Incorporation of label from metabolism of [1,6-^13^C]glucose into newly synthesized lactate (LAC C3) and alanine (ALA C3) in brain from adult WT (white bars) and SIRT3 KO mice (gray bars). Values are mean ± SEM nmol ^13^C incorporated/mg protein from n = 9 WT and 8 SIRT3 KO mice. Individual data points are indicated by circles and triangles. Data were analyzed by Student’s t-test; no differences were found.

The incorporation of label from the metabolism of [1,6-^13^C]glucose into lactate via glycolysis (LAC C3) was not different in SIRT3 KO and WT brain, 12.04 ± 1.37 and 13.83 ± 1.84, respectively, t(15) = 0.763, *p* = 0.456, suggesting that glycolysis was not significantly altered (Figure 3). Labeling of alanine (ALA C3) formed by transamination of the pyruvate generated via glycolysis was also not different in SIRT3 KO when compared to WT mice; the values were 0.80 ± 0.11 and 0.88 ± 0.11 nmol/mg protein, respectively; t(15) = 0.54, *p* = 0.596.

### 3.4. Mitochondrial Oxidative Metabolism Is Decreased in SIRT3 KO Brain

**Figure 4:**
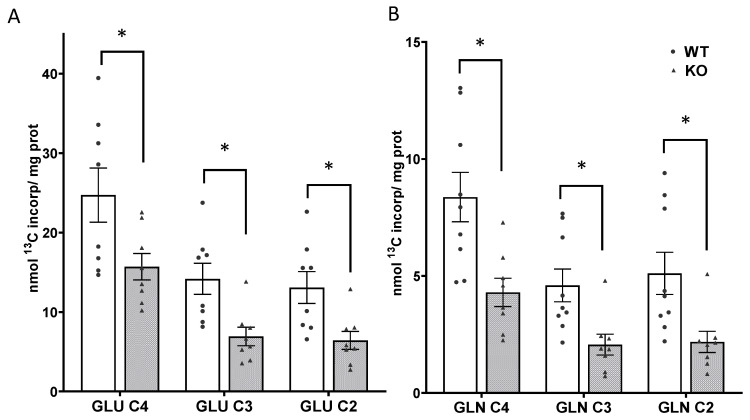
Incorporation of label from metabolism of [1,6-^13^C]glucose into newly synthesized isotopomers of glutamate (**A**) and glutamine (**B**) in brain from adult WT (white bars) and SIRT3 KO mice (gray bars). Individual data points are indicated by black circles and triangles. Note that the scale of the y-axis for glutamine is different than glutamate. Values are mean ± SEM nmol ^13^C incorporated/mg protein from n = 9 WT and 8 SIRT3 KO mice. Data were analyzed by Student’s *t*-test, * *p* < 0.05.

As indicated in the labeling pattern in Figure 1, newly synthesized glutamate labeled from the metabolism of [1,6-^13^C]glucose is formed from the TCA cycle intermediate α-ketoglutarate. There was significantly less incorporation of label into the isotopomers (isotope isomers) of glutamate in SIRT3 KO brain compared to WT mice (Figure 4A). Within the short timeframe of this bolus injection ex vivo NMR study, labeling in GLU C4, which is synthesized primarily from the neuronal TCA and also in astrocytes, in SIRT3 KO mice was 64% of WT; the values were 15.71 ± 1.67 and 24.62 ± 3.00 nmol ^13^C of incorporated/mg protein, respectively, t(15) = 2.504, *p* = 0.024. Labeling in GLU C3 and GLU C2 was lower in SIRT3 KO brain compared to WT mice, t(15) = 3.168, *p* = 0.006 and t(15) = 2.89, *p* = 0.011, respectively.

**Figure 5:**
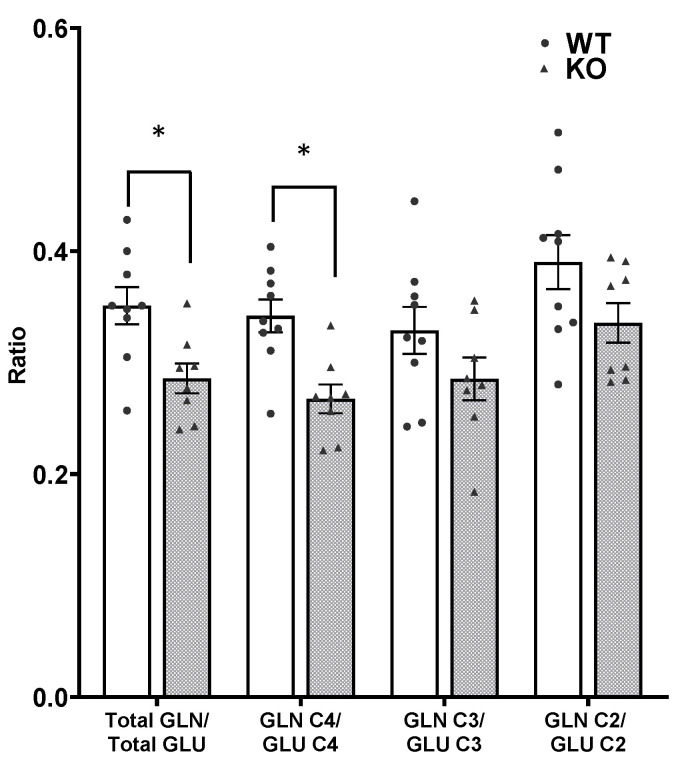
Ratio of newly synthesized glutamine to glutamate in brain from adult WT (white bars) and SIRT3 KO mice (gray bars). Individual data points are indicated by black circles and triangles. Values are mean ± SEM for 9 WT and 8 SIRT3 KO mice. Data were analyzed by Student’s *t*-test, * *p* < 0.05.

There was significantly less incorporation of label into all isotopomers of glutamine in SIRT3 KO brain compared to WT mice (Figure 4B). In the timeframe of this bolus injection, the labeling in GLN C4 reflects synthesis primarily from GLU C4 released by neurons, subsequently taken up by astrocytes and converted to glutamine by the glial enzyme glutamine synthetase. Labeling in GLN C4 in SIRT3 KO brain was 51% of the amount in WT brain t(15) = 3.236, *p* = 0.005. Labeling in GLN C2 reflects labeling via the pyruvate carboxylase (PC) pathway in astrocytes as well as some labeling in neurons. Labeling in GLN C3 reflects labeling from the second turn of the TCA cycle in neurons as well as some labeling from the PC pathway. Labeling in GLN C3 and GLN C2 in the brain from SIRT3 KO mice was 45% and 43% the amount in WT brain, t(15) = 2.96, *p* = 0.009 and t(15) = 2.786, *p* = 0.013, respectively. The PC/PDH ratio, which is calculated from the labeling in the different positions of glutamine (GLN C2-GLN C3)/GLN C4, is an indication of the relative formation of glutamine via the PC pathway in astrocytes [37]. The ratio was 0.04 ± 0.04 and 0.05 ± 0.03 in SIRT3 KO and WT brain, respectively; t(15) = 0.30, *p* = 0.768. The ratios of total labeling in glutamine/glutamate and GLN C4/GLU C4 were lower in brains from SIRT3 KO compared to WT mice (Figure 5), t(15) = 2.985, *p* = 0.009 and t(15) = 3.747, *p* = 0.001, for SIRT3 KO and WT brain, respectively.

**Figure 6:**
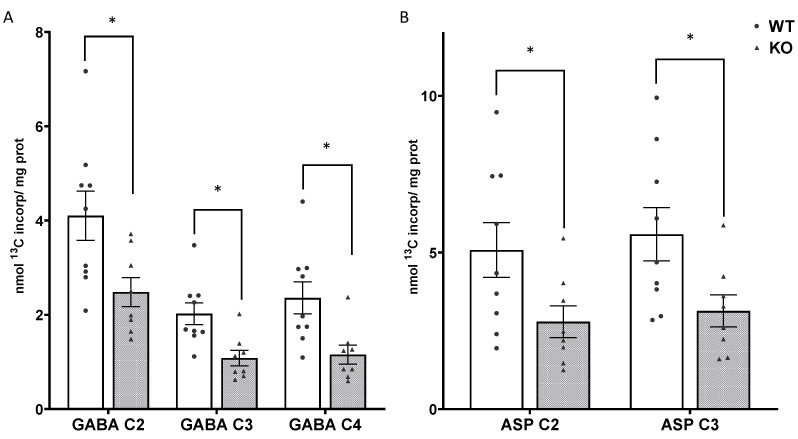
Incorporation of label from metabolism of [1,6-^13^C]glucose into newly synthesized isotopomers of GABA (**A**) and aspartate (ASP) (**B**) in brain from adult WT (white bars) and SIRT3 KO mice (gray bars). Individual data points are indicated by black circles and triangles. Values are mean ± SEM for 9 WT and 8 SIRT3 KO mice. Data were analyzed by Student’s *t*-test, * *p* < 0.05.

There was significantly less incorporation of label into the isotopomers of GABA in SIRT3 KO brain compared to WT mice (Figure 6A). Labeling in GABA C2, which primarily reflects direct synthesis from GLU C4 in neurons in the short timeframe of this study, in SIRT3 KO brain was 64% of the amount in WT brain, t(15) = 2.585, *p* = 0.02. Labeling in GABA C4 reflects GABA primarily from precursors formed via the PC pathway in astrocytes in the short timeframe of this ex vivo study; whereas, labeling in GABA C3 reflects labeling from both neuronal and astrocytic precursors. Labeling in GABA C3 and GABA C4 in SIRT3 KO brain was 53% and 49% of the amount in WT brain, t(15) = 3.232, *p* = 0.005 and t(15) = 2.935, *p* = 0.01, respectively.

### 3.5. Cycling Ratios

Cycling ratios, which are calculated from the labeling from the second turn of the TCA cycle relative to the labeling from the first turn of the cycle (e.g., GLU C3/GLU C4) have been used as an indication of the turnover of a metabolite (e.g., glutamate) in the TCA cycle. Ratios were decreased for glutamate and GABA in the brain from SIRT3 KO mice compared to WT, suggesting slower turnover of metabolism in both glutamatergic and GABAergic neurons. The cycling ratios for glutamate was 0.43 ± 0.04 in the brain from SIRT3 KO mice and 0.56 ± 0.02 in WT brain, respectively; t(15) = 3.524, *p* = 0.009. The cycling ratio for GABA was 0.43 ± 0.04 and 0.50 ± 0.02 in brains from SIRT3 KO mice compared to WT brain, respectively, t(15) = 2.732, *p* = 0.015. In contrast, the cycling ratio for glutamine was not different; the values were 0.46 ± 0.05 and 0.54 ± 0.03 in the brain from SIRT3 KO mice compared to WT brain, respectively, t(15) = 1.78, *p* = 0.09.

There was significantly less incorporation of label from the metabolism of [1,6-^13^C]glucose into the C3 and C2 positions of aspartate. Labeling in ASP C3 and ASP C2 in SIRT3 KO brain was 56% and 55% of the level in the brain of WT mice (Figure 6B); t(15) = 2.393, *p* = 0.03, and t(15) = 2.195, *p* = 0.044, respectively. The difference in labeling in ASP C3 and ASP C2 can be used to calculate the anaplerotic ratio for synthesis of aspartate via the PC pathway in astrocytes. Despite the large difference in labeling, the anaplerotic ratio for aspartate (ASP C3-ASP C2)/ASP C2 was comparable in brains from SIRT3 KO and WT mice. Values were 0.16 ± 0.09 and 0.16 ± 0.08 in the brain from SIRT3 KO and WT mice, respectively.

### 3.6. Metabolism via the TCA Cycle and the Pyruvate Recycling Pathway

The incorporation of label from the metabolism of [1,6-^13^C]glucose into the C3 and C2 positions of the symmetrical TCA cycle intermediate succinate (Succ C2/3) was not different in SIRT3 KO mice and WT mice. Values were 0.70 ± 0.11 and 0.99 ± 0.13 nmol ^13^C incorporated/mg protein, respectively; t(15) = 1.768, *p* = 0.09. Labeling of the C2 position of lactate, which occurs only via the pyruvate recycling pathway was the same in SIRT3 KO and WT brain. Values were 0.62 ± 0.08 and 0.60 ± 0.11 nmol ^13^C incorporated/mg protein.

### 3.7. Percent Enrichment of Metabolites

The percent enrichment of a specific isotopomer (e.g., Glutamine C2) indicates how much of the isotopomer is labeled relative to the total pool of that particular metabolite (e.g., total glutamine pool). Percent enrichment was calculated for all of the metabolites determined by ^13^C-NMR (Table 1).

There was no difference in the percent enrichment in LAC C3 and ALA C3 in the brain from SIRT3 KO and WT mice, consistent with the similar labeling via glycolysis in the KO and WT brain. The enrichment of GLU C3 and GLU C2 was significantly lower in the brain from SIRT3 KO compared to WT mice (Table 1). Since the total glutamate pool size was not different in SIRT3 KO and WT brain (Figure 2), the lower enrichment reflects less synthesis of glutamate in KO brain.

The enrichment of GLN C3 and GLN C2 was lower in the brain from SIRT3 KO compared to WT mice, and enrichment in GLN C4 tended to be lower in SIRT3 KO brain. Although the concentration of glutamine and labeling of newly synthesized GLN C3 and GLN C2 were lower in brains from SIRT3 KO mice (Figure 4A,B), the decrease in GLN enrichment is due to more of an effect on the synthesis of glutamine than on the total GLN pool in the brain of KO mice. The enrichment in isotopomers of GABA was not different in brains from SIRT3 KO compared to WT mice. This reflects the combined effect of lower synthesis and the smaller total GABA pool in the brains of SIRT3 KO compared to WT mice. The enrichment of ASP C2 and ASP C3 were not different in the brain from SIRT3 KO compared to WT mice. The enrichment of the TCA cycle intermediate SUCC C2/3 and LAC C2, which is labeled via metabolism in the pyruvate recycling pathway were not different in brains from SIRT3 KO compared to WT mice.

## 4. Discussion

SIRT3 deficient mice are viable under both fed and fasted conditions, and SIRT3 deficiency does not affect mitochondrial content or blood glucose levels [12,28,38]. Our findings that the total glucose concentration and amount of unmetabolized ^13^C-glucose in the brain were not different in SIRT3 KO and WT mice, demonstrates that *Sirt3* deficiency does not affect glucose uptake into the brain.

There are conflicting reports in the literature regarding the intracellular SIRT3 localization. It was reported that SIRT3 is processed in the nucleus prior to translocation to mitochondria only under stress conditions [39], whereas another group reported that the long inactive form is processed in the mitochondria to the active short form [40]. We and other laboratories showed that SIRT3 is present exclusively in mitochondria in the brain or liver of naïve wild type mice [19,40,41], suggesting that SIRT3 is localized to brain mitochondria under normal, physiologic conditions.

Since the acetylation of mitochondrial proteins in mouse brain is controlled by SIRT3, the absence of this enzyme activity leads to a dramatic increase in acetylation of mitochondrial proteins including enzymes involved in glucose metabolism, particularly TCA cycle proteins, respiratory chain enzymes, and enzymes of amino acid metabolism [42,43,44,45]. Thus, cells with disrupted *sirt3* gene show a profound increase in mitochondrial protein acetylation, leading to major defects in oxidative glucose metabolism [42].

In this study, we determined the overall brain glucose metabolism in SIRT3 KO mice, which is, for the reasons noted above, essentially an examination of the impact of increased mitochondrial protein acetylation on brain glucose metabolism. Since the SIRT3 activity is NAD^+^ dependent, a significant reduction in the total mitochondrial NAD^+^ pool leads to mitochondrial protein hyperacetylation [27], thus causing a similar effect as knockout/disruption of the *sirt3* gene. Therefore, the observed changes in glucose metabolism reported here might parallel alterations in glucose metabolism under several disease conditions that are associated with a dramatic decrease in mitochondrial NAD^+^ levels. For example, there is a reduction in mitochondrial NAD^+^ content following global cerebral ischemia [27,46,47,48], and NAD^+^ levels decline with aging [49]. Conversely, administration of the NAD^+^ precursor nicotinamide mononucleotide (NMN) resulted in improved energy metabolism in a Parkinson’s disease model [50] and also ameliorated the mitochondrial respiratory deficits in an Alzheimer’s disease-relevant murine model [51].

### 4.1. Sirt3 Does Not Affect Glucose Uptake or Glycolytic Metabolism

As expected, the deletion of the *sirt3* gene did not affect the ^13^C glucose transport into the brain since 15 min after the i.p. injection of [1,6-^13^C]glucose the concentration of unmetabolized brain glucose, and the incorporation of label into newly synthesized LAC C3 was not different in WT and SIRT3 KO animals. Correspondingly, the concentration of lactate and alanine did not differ between the WT and SIRT3 KO brains, suggesting that the glycolytic pathway was not significantly affected in Sirt3 KO brain. This is in agreement with reports showing no effect of SIRT3 on cytosolic enzymes or SIRT1 and SIRT2 expression levels or activity [52].

The end product of glycolysis, pyruvate, can be either converted to lactate by lactate dehydrogenase (LDH) or to alanine by transamination. Pyruvate is also transported into mitochondria where it supplies carbon in the form of acetyl CoA to the TCA cycle via activity of PDH in neurons and astrocytes. In addition, pyruvate can be converted to the TCA cycle intermediate oxaloacetate by PC in astrocytes. The present study found no impact of SIRT3 KO on the brain glycolytic pathway since the incorporation of label from glucose metabolism into newly synthesized lactate (LAC 3) and alanine (ALA C3) was the same in SIRT3 KO and WT brain. This is consistent with the localization of SIRT3 in the mitochondrial matrix, suggesting that SIRT3 deacetylase has no ability to directly change the acetylation status of cytosolic proteins participating in glycolysis [13,23].

### 4.2. Differential Incorporation of ^13^C into Metabolites in Wild Type and SIRT3 KO Mice

**Figure 7:**
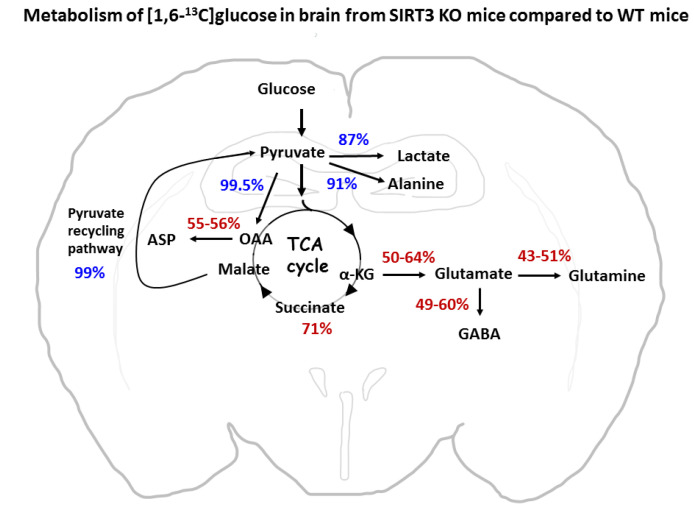
Schematic of metabolism in brain from SIRT3 KO mice compared to WT mice. Numbers represent percent of metabolism in brain from SIRT3 KO mice compared to WT mice. The percent metabolism is calculated from the data in Figure 2, Figure 3, Figure 4, Figure 5 and Figure 6. A range is indicated where there is a difference in labeling of the different isotopomers of the compound. Numbers in blue indicate pathways that are not different in SIRT3 KO and WT brain. Numbers in red show pathways that are significantly decreased in brain from SIRT3 KO mice compared to WT mice. Abbreviations: ASP, aspartate; α-KG, α-ketoglutarate; OAA, oxaloacetate.

Although the uptake of labeled glucose and incorporation of label into lactate and alanine were not changed in the brain from SIRT3 KO mice when compared to WT animals, there was a significantly lower incorporation of label into all isotopomers of glutamate (GLU C4, GLU C3, and GLU C2) and glutamine (GLN C4, GLN C3, and GLN C2) in animals with disrupted *sirt3* gene. These data indicate impaired oxidative metabolism in SIRT3 KO brain. Reports showing inhibition of enzymes activity downstream of pyruvate metabolism (PDH and in the TCA cycle) by increased acetylation (see [3,12,29]) offer one possible cause for the disturbed mitochondrial glucose metabolism. The overall effect of deletion of the *sirt3* gene on the metabolism of glucose in this study is summarized in Figure 7. The incorporation of label into newly synthesized GLU C4 can be affected by the activity of pyruvate dehydrogenase (PDH) and several enzymes of the TCA cycle. Pyruvate is converted to α-ketoglutarate (α-KG) through coupled reactions of pyruvate dehydrogenase (PDH), citrate synthase (CS), mitochondrial aconitase 2 (ACO2), and mitochondrial isocitrate dehydrogenase 2 (ICDH 2). The GLU C4 is formed from labeled α-KG in the mitochondrial matrix, which is converted to glutamate (GLU C4) primarily by the transamination activity of aspartate aminotransferase (AAT/GOT), and to a lesser extent by alanine transaminase (ALT), and branched chain amino acid transaminase (BCAT) and also by glutamate dehydrogenase (GDH).

Pyruvate formed via glycolysis is transported into the mitochondria by the mitochondrial pyruvate carrier (MPC) [53,54]. Increased acetylation-induced destabilization of the MPC in the absence of *Sirt3* [55] has been shown to impair uptake of pyruvate into the mitochondria. However, from the present study it is not known if alterations in pyruvate uptake into mitochondria contributed to the changes in metabolism. The activity of PDH can be reduced in the absence of SIRT3 activity due to hyperacetylation of E1a subunit [22]. Similarly, the hyperacetylation of CS and ICDH2 leads to inhibition of enzyme activity [56,57]. Although aconitase is activated by acetylation [58,59], this enzyme is highly sensitive to free radical-induced damage [60,61,62,63]. Thus, it is likely that aconitase activity is also suppressed in *Sirt3* KO brain since mitochondrial superoxide production is significantly increased due to increased acetylation-induced inhibition of mitochondrial superoxide dismutase in SIRT3 KO animals [27,64]. All the above-mentioned TCA cycle enzymes, except aconitase, are inhibited by increased acetylation that would occur in the brain of SIRT3 KO mice [3,12,22,29,56,57]. Similarly, since GLU C3 and some of the GLU C2 are generated from metabolism in the second turn of the TCA cycle, the acetylation-induced inhibition of the above listed enzymes, in conjunction with inhibition of succinate dehydrogenate (SDH) by acetylation [65] that is downstream of α-KG, would lead to lower incorporation of label into GLU C3 and GLU C2. The activity of PDH is much higher in neurons than in astrocytes, therefore these changes in glucose metabolism would primarily reflect an inhibition of neuronal mitochondrial TCA cycle activity. The reported findings in the literature regarding the inhibitory effect of acetylation on mitochondrial enzymes activity are in agreement with our data showing both reduced labeling of glutamate isotopomers and the lower percent enrichment of GLU C3 and GLU C2 in the total pool of glutamate, resulting from suppressed downstream glucose metabolism in both neurons and astrocytes.

In interneurons, glutamate serves as substrate for glutamate decarboxylase (GAD), the enzyme that produces GABA. The significantly reduced incorporation of label into newly synthesized isotopomers of GABA (GABA C4, GABA C3, and GABA C2) also suggests the lower availability of newly synthesized glutamate in SIRT3 KO animals, since to our knowledge acetylation of GAD had not been reported. The possibility of slower mitochondrial metabolism due to lower expression levels of TCA cycle enzymes is unlikely since only the expression level of isocitrate dehydrogenase 2 (IDH2) was reported to be reduced in SIRT3 KO animals [43], and hyperacetylation of this enzyme due to the absence of SIRT3 activity is known to be inhibitory [66,67].

### 4.3. Perturbed Metabolic Coupling between Neurons and Astrocytes in SIRT3 KO Brain

Glutamate released from neurons is taken up by astrocytes and converted to glutamine by glutamine synthetase (GS) or oxidized for energy [68]. When acetylated, GS interacts with cullin-RING ubiquitin ligase 4 (CRL4), gets ubiquitinated and degraded by the proteasome [69]. However, GS is located in cytoplasm and therefore GS acetylation is not affected by decreased SIRT3 activity. This implies that the lower incorporation of label into GLN C4, GLN C3 and GLN C2 is mainly determined by limited availability of GLU C4, GLU C3, GLU C2 released by neurons, or decreased de novo formation of GLU C2 and GLU C3 via the PC pathway in astrocytes in SIRT3 KO animals. This is also reflected in the reduction in the total glutamine pool in the SIRT3 KO brain when compared to the WT brain. However, it should be noted that Gonzalez Herrera et al. [70] showed that loss of SIRT3 led to the increased use of glutamine in nucleotide biosynthesis in embryonic stem cells, which limited the use of glutamine in other pathways. It is not known if this occurs in the brain of adult SIRT3 KO mice.

In astrocytes, pyruvate metabolism is linked to the TCA cycle via pyruvate carboxylase (PC), which generates oxaloacetate (OAA). OAA also serves as a substrate for aspartate amino transferase (AAT), leading to production of aspartate. In SIRT3 KO brain the incorporation of label into aspartate isotopomers (ASP C3 and ASP C2) is reduced by about 55% when compared to WT brain. Although acetylation of PC is modulated by SIRT3 [20,43], it is not known whether the acetylation has an inhibitory or stimulatory effect on the enzyme activity. A study by Yang et al. shows that AAT can be acetylated at three lysine residues. The acetylation enhances the association between mitochondrial AAT and malate dehydrogenase (MDH), leading to stimulation of the malate-aspartate shuttle [71]. Although the lower incorporation of the label into ASP C3 and ASP C2 might be due in part to decreased activity of astrocytic PC in SIRT3 KO animals, the finding that the anaplerotic ratio for aspartate was not significantly different argues against this possibility. The lack of a difference in the PC/PDH ratio for glutamine in SIRT3 KO and WT mice is consistent with impairment in both astrocyte and neuronal metabolism in KO brain. Thus, metabolism in the TCA cycle, which also leads to labeling in ASP C3 and C2 (Figure 1) may contribute more to the lower labeling of aspartate in the brain. This possibility would suggest a major contribution from impaired neuronal metabolism, and less of a contribution from astrocyte metabolism, in SIRT3 KO brain compared to WT brain.

Taken together, our data provide evidence of impaired mitochondrial glucose metabolism, which is assumed to be due to a significant reduction in the activity of mitochondrial enzymes that are involved in bioenergetic metabolism in SIRT3 KO brain (see Figure 7). Overall, these changes in metabolism would lead to the reduced ability of mitochondria to generate sufficient ATP, particularly under stress conditions. This is in agreement with findings showing that the baseline ATP levels in SIRT3 KO animals are significantly reduced in the brain [4], probably also due to reduced ATP synthase activity [24]. The decreased total creatine in brains from SIRT3 KO compared to WT mice also supports this concept. Although a lower brain ATP level was reported in SIRT3 KO animals by one group [4], others did not report differences in ATP levels between WT and SIRT3 KO mice, particularly in liver and muscle tissue [38]. Furthermore, SIRT3 KO mice display similar overall energy balance in both the fed and fasted states as WT littermates under non-stress conditions [28]. These reports suggest that loss of SIRT3 activity may have a variable effect on metabolism in different organs and cell types.

In conclusion, the hyperacetylation of mitochondrial enzymes due to the absence of SIRT3 activity compromises glucose metabolism preferentially at the mitochondrial TCA cycle enzymes, resulting in impaired generation of downstream metabolites including glutamate, glutamine, aspartate and GABA. Although, the mitochondrial bioenergetic metabolism is compromised in the SIRT3 KO animals, to date, it has not been shown to lead to brain dysfunction or damage under physiologic conditions. However, hyperacetylation of mitochondrial proteins represents an aggravating factor that can significantly compromise cell survival under stress, and thus contribute to the pathologic mechanisms leading to cell death.

## Figures and Tables

**Table 1:** Enrichment of newly synthesized metabolites in the brain from SIRT3 KO and WT mice.

Metabolite	WT % Enrichment	SIRT3 KO % Enrichment	Significance
Glucose	38.62 ± 7.23	37.98 ± 4.33	*t* = −0.074, *p* = 0.942
Lactate C3	28.25 ± 3.55	26.24 ± 3.45	*t* = 0.423, *p* = 0.673
Alanine C3	36.19 ± 5.16	33.26 ± 5.15	*t* = 0.451, *p* = 0.659
Glutamate C4	32.40 ± 3.88	23.02 ± 3.15	*t* = 1.847, *p* = 0.084
Glutamate C3	18.15 ± 2.20	10.23 ± 1.93	*t* = 2.669, *p* = 0.017
Glutamate C2	16.69 ± 2.23	9.53 ± 1.88	*t* = 2.419, *p* = 0.028
Glutamine C4	23.18 ± 2.22	16.26 ± 2.38	*t* = 2.12, *p* = 0.050
Glutamine C3	12.67 ± 1.56	7.87 ± 1.68	*t* = 2.09, *p* = 0.053
Glutamine C2	14.00 ± 2.14	8.32 ± 1.73	*t* = 2.02, *p* = 0.061
GABA C2	25.90 ± 2.49	21.55 ± 3.26	*t* = 1.076, *p* = 0.299
GABA C3	12.92 ± 1.24	9.40 ± 1.61	*t* = 1.752, *p* = 0.100
GABA C4	14.75 ± 1.56	10.06 ± 1.93	*t* = 1.901, *p* = 0.076
Lactate C2	1.37 ± 0.14	1.34 ± 0.17	*t* = 0.15, *p* = 0.882
Aspartate C2	27.51 ± 3.99	18.14 ± 3.75	*t* = 1.69, *p* = 0.110
Aspartate C3	30.62 ± 3.73	20.15 ± 3.76	*t* = 1.97, *p* = 0.067
Succinate C2/3	26.64 ± 3.32	20.68 ± 3.39	*t* = 1.253, *p* = 0.229

Data were analyzed by paired Students *t*-tests to determine differences in enrichment in the brain from SIRT3 KO and WT mice. *n* = 8 for SIRT3 KO and 9 for WT brains.

## Data Availability

The data presented in this study are available on request from the corresponding author. The data are not publicly available.
